# Public health round-up

**DOI:** 10.2471/BLT.19.010619

**Published:** 2019-06-01

**Ebola response**

A health worker administers Ebola vaccine to a patient at the Beni Ebola treatment centre in North Kivu in the Democratic Republic of the Congo.

**Figure:**
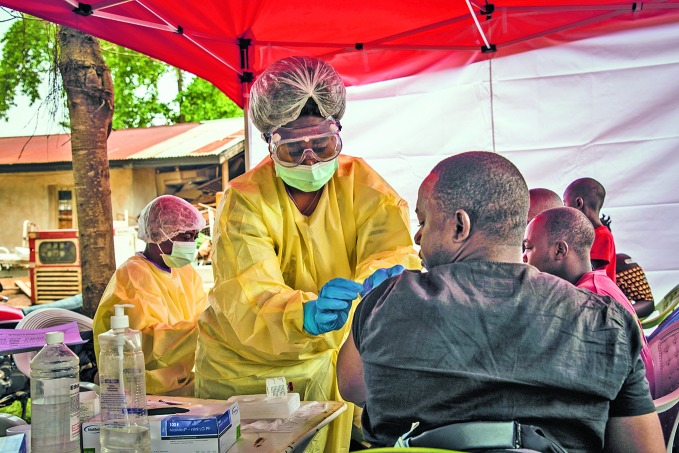
World Bank/Vincent Tremeau

## Ebola response hampered by violence

Ebola outbreak response activities, including vaccination, infection prevention and control and surveillance, in Butembo, the Democratic Republic of the Congo were suspended for several days in early May after a series of violent incidents. 

The incidents included an attack on the city centre by 50 militia members on 8 May. The attack was repelled by security forces in an intense exchange of gunfire close to World Health Organization (WHO) staff accommodation. Ebola epidemic response activities had partially resumed by 9 May.

The incidents followed the 19 April attack on Butembo University Hospital in which Richard Valery Mouzoko Kiboung died from a gunshot wound, sustained while chairing a meeting with health workers.

WHO Director-General Tedros Adhanom Ghebreyesus and WHO Regional Director for Africa, Matshidiso Moeti, visited Butembo on 30 April to show support to WHO and partner organization staff, and to discuss how to strengthen security and the Ebola response effort. They also met local political, business and religious leaders and called on them to accelerate their efforts to stabilize the surrounding environment. “Some would have Ebola drive us apart. We can only defeat it if we all work together,” Moeti said.

The Director-General and Regional Director urged the international community to step up support to contain the Ebola outbreak, including filling the funding gap that threatens to compromise the Ebola response.

Ebola virus transmission remains focused on the Beni, Butembo, Mabalako, Mandima, Musienene, Kalunguta and Katwa health zones, which together account for 93% of the 303 cases reported between 17 April and 7 May. As of 11 May, a total of 1680 confirmed and probable Ebola cases had been reported, and 1117 people had died.

https://www.who.int/csr/don/09-may-2019-ebola-drc/en/

## Ebola vaccination adjustments

WHO’s Strategic Advisory Group of Experts issued new recommendations to address vaccination challenges in the Ebola outbreak in the DRC.

Published 7 May, the recommendations include implementing dosage adjustments based on available efficacy data, expansion of population coverage of rVSV-ZEBOV-GP vaccination and operational adjustments to expedite the vaccination process. 

The group also recommended introducing an additional investigational vaccine, and stepping up efforts to train nurses, doctors and medical students from Ebola-affected communities to work on vaccination teams.

More than 114 000  people have been vaccinated in the central African country since the outbreak was declared in August 2018.

https://www.who.int/immunization/policy/position_papers/interim_ebola_recommendations_may_2019.pdf?ua=1&ua=1

## First malaria vaccine launched

Malawi and Ghana rolled out the world’s first malaria vaccine in April, the first African countries to do so. Kenya was scheduled to roll out its vaccination campaign in the second half of May. Known as RTS,S, the vaccine will be made available for children under 2 years of age.

In clinical trials, the vaccine was found to prevent approximately 4 in 10 malaria cases, including 3 in 10 cases of severe malaria. The vaccine was developed over three decades by pharmaceutical company GlaxoSmithKline in collaboration with PATH’s Malaria Vaccine Initiative and a network of African research centres.

Malaria is one of the world’s leading infectious disease killers, with most malaria deaths occurring in Africa. Children under 5 years are at greatest risk of malaria’s life-threatening complications. Worldwide, the disease kills an estimated 435 000 people a year, two-thirds of them children.

https://www.afro.who.int/news/malaria-vaccine-pilot-launched-ghana

## Cutting out trans fat

The International Food and Beverage Alliance announced plans to ensure that the amount of industrial trans fat in their products does not exceed 2 g per 100 g fat or oil by 2023.

Announced on 7 May, the plans are in line with the recommendations of WHO’s REPLACE action package, which calls for all food producers, including oil and fat manufacturers, to commit to eliminating industrial trans fat from the global food supply.

Trans fat derives from natural and manufactured sources. Consuming trans fat raises low-density lipoprotein cholesterol levels while lowering high-density lipoprotein cholesterol levels. Trans fat intake is responsible for more than 500 000 deaths from coronary heart disease each year worldwide.

https://www.who.int/docs/default-source/documents/replace-transfats/replace-action-package.pdf

## Call for action on antimicrobial resistance

The United Nations ad hoc Interagency Coordinating Group released a report calling for immediate action to address the growing challenge posed by antimicrobial resistance.

The report, entitled, *No time to wait: securing the future from drug-resistant infections*, advocates a One Health approach supported by international organizations, governments and other stakeholders.

The report, released on 29 April, estimates that antimicrobial resistance will lead to 10 million deaths each year by 2050. Currently, it is estimated that around 700 000 people die each year due to drug-resistant infections, including 230 000 people who die from drug-resistant tuberculosis.

https://www.who.int/antimicrobial-resistance/interagency-coordination-group/final-report/en/

## WHO guidance on under-5 activity

Children under five years of age should spend less time staring at screens and more time being physically active, according to new WHO guidelines.

Published 24 April, *Guidelines on physical activity, sedentary behaviour and sleep for children under 5 years of age* was developed by a WHO panel of external independent experts, who assessed the effects on young children of inadequate sleep, and of time spent sitting down and watching screens. 

The panel also reviewed the evidence for benefits of increased activity levels.

https://apps.who.int/iris/handle/10665/311664

**Cover photo**

A young child stands in a doorway in Beni, one of the cities that has been affected by the Ebola outbreak in the Democratic Republic of the Congo.

**Figure:**
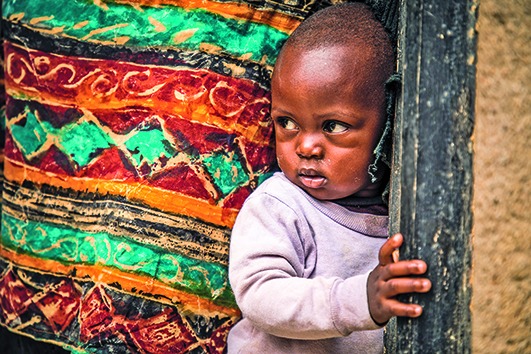
World Bank/Vincent Tremeau

## New snakebite strategy

A new strategy aimed at halving the number of deaths and cases of disability due to snakebite envenoming over the next 12 years was launched by the governments of Costa Rica and Nigeria on 23 May in Geneva, Switzerland.

The strategy, entitled *Snakebite envenoming: a strategy for prevention and control,* is designed to support efforts to prevent and control snakebite envenoming, and stresses the importance of community education and empowerment, effective first response, and ensuring access to safe, effective treatment, such as antivenoms and ancillary medical care.

Snakebite envenoming affects 1.8–2.7 million people each year, claiming 81 000–138 000 lives and causing 400 000 cases of permanent disability.

https://apps.who.int/iris/bitstream/handle/10665/312195/WHO-CDS-NTD-NZD-2019.03-eng.pdf?ua=1

## First digital health guidelines

WHO released its first guideline on digital health interventions on 17 April. The guideline focuses on digital health interventions that are primarily available via mobile devices and can support progress towards universal health coverage.

https://apps.who.int/iris/bitstream/handle/10665/311977/WHO-RHR-19.8-eng.pdf?ua=1

## Dementia guidelines

WHO launched guidelines aimed at reducing the risk of cognitive decline and dementia on 14 May. The guidelines provide recommendations on behaviours and on interventions to delay or reduce cognitive decline and dementia in the general population.

The guidelines are aimed at health-care providers, policy-makers, health-care planners and programme managers, and provide a knowledge base that Member States can use to develop their national responses to address this challenge.

Dementia is a rapidly growing public health problem affecting around 50 million people worldwide. There are nearly 10 million new cases every year and this figure is set to triple by 2050. 

https://www.who.int/mental_health/neurology/dementia/guidelines_risk_reduction/en/

**Looking ahead**

14 – 17 July - Sexually transmitted infections and human immunodeficiency virus 2019 World Congress, Vancouver, Canada

23 September – United Nations High-Level Meeting on universal health coverage. UN Headquarters, New York, United States of America.

24 – 25 September - Sustainable Development Goals Summit, New York, United States of America.

